# Behavioral factors associated with HCV and HIV co-infection in residents of São Paulo, Brazil

**DOI:** 10.1186/1742-4690-9-S1-P130

**Published:** 2012-05-25

**Authors:** Norma Farias, Umbeliana Barbosa de Oliveira, Iára de Souza, Débora Moraes Coelho, Claudia Afonso Binelli

**Affiliations:** 1 State Secretary of Health of São Paulo, São Paulo, Brazil

## Introduction

To date, there are no surveys in Brazil on the occurrence of co-infection hepatitis and HIV in the general population. The aim of the present study was to investigate factors associated with HIV/HCV co-infection among residents in the State of Sao Paulo, Brazil, notified at the National Databank of Major Causes of Morbidity.

## Material and methods

We reviewed 3,032 cases of HIV/HCV co-infection among 46,969 bank records of viral hepatitis from January 2007 to March 2010. The hepatitis C cases were confirmed by the presence of HCV RNA using reverse transcription-polymerase chain reaction (RT-PCR) in anti-HCV-positive samples. The diagnosis of HIV/AIDS and data on demographic and behavioral aspects were collected through epidemiologic investigation forms. Variables associated with HCV/HIV co-infection were identified with Poisson regression model and confidence intervals of 95%.

## Results

The majority were male (73%), white (65%) and had less than 50 years (65%). In a adjusted analysis, the prevalence ratio was 1.27 (CI 95%:1.04-1.55) for sexual contact with patients with HVB or HCV, 1.48(CI 95%: 1.27-1,73) for 3 or more sexual partners, 1.73 (CI 95%: 1.38-2.16) for STD, 2.95 (95% CI:2.42-3.59) among IDU and 1.78(95% CI:1.49-2.17) among inhalable drug users or crack.

## Conclusions

These data show that illicit drug use is the main factor associated with co-infection HCV / HIV in São Paulo. Sexual transmission suggested to play a role in co-infected HCV / HIV.

